# A retrospective study of cytomegalovirus pneumonia in renal transplant patients

**DOI:** 10.3892/etm.2014.1577

**Published:** 2014-02-24

**Authors:** BIAO DONG, YUANTAO WANG, GANG WANG, WEIGANG WANG, HONGLAN ZHOU, YAOWEN FU

**Affiliations:** Department of Urology, The First Hospital of Jilin University, Changchun, Jilin 130021, P.R. China

**Keywords:** kidney transplantation, cytomegalovirus pneumonia, glucocorticosteroid, immunosuppressants, induction therapy

## Abstract

The aim of the present study was to investigate an optimal prophylaxis of cytomegalovirus (CMV) pneumonia in renal transplant recipients. A total of 83 kidney transplant recipients who had been diagnosed with CMV pneumonia between January 2008 and December 2011 were enrolled in the study. Patients were assigned to a standard or improved group based on the prophylaxis administered. The retrospective study was undertaken to compare the incidence of CMV pneumonia, cure rate or recovery rate and mortality between the two groups. The results indicated that a longer duration of prophylaxis with oral ganciclovir effectively reduced the risk of CMV pneumonia in kidney transplant recipients. Treatments, including early withdrawal of immunosuppressants, regular use of glucocorticosteroids and careful supportive therapy, were beneficial in controlling CMV pneumonia. Furthermore, antibody induction therapy may not increase the risk of CMV pneumonia in kidney recipients administered proper prophylaxis [3-month course of oral ganciclovir and trimethoprim-sulfamethoxazole (SMZ-TMP)]. Therefore, the present study demonstrated that a longer duration of prophylaxis with oral ganciclovir, withdrawal of immunosuppressants and regular use of glucocorticosteroids may be improved treatments for CMV pneumonia.

## Introduction

Cytomegalovirus (CMV) infection occurs in the majority of solid organ transplant recipients, primarily in the first three to six months following transplantation when immunosuppressants are intensely administered (1). Relentlessly progressive interstitial pneumonia is primarily associated with CMV infection following kidney transplantation and develops into acute respiratory distress syndrome, usually leading to respiratory failure and mortality. CMV pneumonia is a common cause of mortality in renal transplant recipients with a reported fatality rate of 65–90% (2). Therefore, early diagnosis, strategic prevention and active treatment of CMV pneumonia are crucial for kidney transplant success.

Ganciclovir, a nucleoside analogue that inhibits DNA synthesis, is an antiviral medication used to prevent or treat CMV infections. Prophylaxis with high doses of intravenous or oral ganciclovir reduces the risk of CMV infection following organ transplantation (3). A previous study demonstrated that preemptively administered oral ganciclovir was as effective as intravenous ganciclovir for treating CMV infection following liver transplantation (4).

CMV-induced pulmonary lesions exhibit diffuse alveolar damage and/or interstitial inflammation. Glucocorticoids suppress the release of alveolar effusion fluids, reduce the inflammatory reaction of lung parenchyma and interstitial tissue and further prevent pulmonary interstitial fibrosis (5,6).

Reducing or even withdrawing immunosuppressive drugs, including calcineurin inhibitors (CNIs), is important at an early stage when the kidney recipients are diagnosed with CMV pneumonia (7).

Induction therapy with polyclonal and monoclonal antibody transplantation has been used for >30 years (8) and is routinely utilized to prevent and treat solid organ rejection. These antibodies may become the crucial factors in the immunosuppressive regimens (9). The percentage of kidney transplant recipients receiving induction therapy is >80%, with the majority receiving antithymocyte globulin (rATG), basiliximab, daclizumab or alemtuzumab, alone or in combination with steroids (10). However, these antibody therapies have been associated with an increased incidence of neoplastic complications and opportunistic infections, particularly CMV infection (11). A study by Luan *et al* (12) indicated that in the absence of prophylaxis, the use of rATG was associated with a higher risk of CMV infection.

The aim of the present retrospective study was to compare the risk of CMV infection among 83 kidney recipients with CMV pneumonia that were divided into two groups; one group that received induction therapy and one group that did not. In addition, the present study investigated an optimal prophylaxis (ganciclovir and glucocorticoid treatment, withdrawal of the immunosuppressive drugs and nutritional support) under which antibody induction therapy did not increase the risk of CMV pneumonia in kidney recipients.

## Patients and methods

### Subjects

A total of 573 kidney transplant recipients were enrolled in the study between January 2008 and December 2011. All the transplanted kidneys were obtained from living-related donors or brain-dead cadavers. The warm and cold ischemia times were 7.4±2.8 min and 9.8±2.9 h, respectively. Among the 205 kidney recipients that were diagnosed with pulmonary infection following transplantation, 83 patients were diagnosed with CMV pneumonia. All the CMV pneumonitis cases occurred with the first kidney transplantation. There were no marked X-ray abnormalities in any recipients prior to transplantation. The matched donors and recipients had the same blood type and shared at least one human leukocyte antigen haplotype (HLA-A, B, DR). Tests for lymphocytotoxicity were negative and the panel reactive antibody score was <10.0%.

### Grouping

A total of 138 patients underwent allograft renal transplantation between January 2008 and December 2008. Pulmonary infection was diagnosed in 65 kidney recipients following transplantation, among whom CMV pneumonia was confirmed in 38 patients according to laboratory tests and medical imaging. The patients with CMV pneumonia in this group received standard treatment and were referred to as the standard group (Group S). An additional 435 patients underwent allograft renal transplantation between January 2009 and December 2011. In total, 45 recipients were confirmed to have CMV pneumonia among the 140 recipients that were diagnosed with pulmonary infection. These patients with CMV pneumonia received improved treatment and were referred to as the improvement group (Group I). There were 38 patients with CMV pneumonia in Group S, including 27 males and 11 females, with ages ranging between 21 and 60 years. There were 45 patients with CMV pneumonia in Group I, including 30 males and 15 females, aged between 24 and 66 years-old.

### Inclusion and exclusion criteria

Inclusion criteria included a regular fever for >3 days and a body temperature of ≥38°C. In addition, patients were included if they exhibited symptoms of chest distress, a dry cough and dyspnea and had X-ray or computed tomography (CT) scans that showed interstitial inflammation changes in the lungs. Patients that were shown to have hypoxemia by blood gas analysis were also included. Finally, serological blood tests were required to be positive for CMV-PP65 antigen, anti-CMV IgG antibody, anti-CMV IgM antibody and CMV-DNA (a 4-fold increase in IgG titers was required if previously negative). However, tests for bacteria, mold, *Mycobacterium tuberculosis*, *Pneumocystis carinii*, Epstein-Barr (EB) virus, mycoplasma and *Chlamydia trachomatis* were negative.

Patients were excluded from the study if they had no fever or if it lasted <3 days or >3 days but had no regularity. Exclusion criteria also included symptoms such as a cough, expectoration and dyspnea. In addition, patients were excluded if X-ray or CT scans did not show interstitial inflammation changes in the lungs. Finally, if the CMV-PP65 antigen test was negative, or if at least one test was positive for bacteria, mold, *Mycobacterium tuberculosis, Pneumocystis carinii*, EB virus, mycoplasma or *Chlamydia trachomatis,* patients were excluded.

### CMV prophylaxis

In Group S, kidney transplant recipients received a 2-week course of intravenous ganciclovir during their hospital stay. Whilst in Group I, kidney transplant recipients received a 3-month course of oral ganciclovir in the post-discharge period. Kidney transplant recipients in the two groups received 0.5 g/day trimethoprim-sulfamethoxazole (SMZ-TMP) for 3 months in the post-discharge period.

### Therapeutic regimen

Patients in Group S, when diagnosed with CMV pneumonia, received a reduced dose of immunosuppressants. In the majority of kidney recipients, the dosage of tacrolimus and ciclosporin A was reduced by one third, while mycophenolate mofetil (MMF) was reduced by a half, with the exception of five patients that were unable to control the CMV infection when the immunosuppressants were completely withdrawn. In addition, oral administration of prednisone was stopped and 40 mg methylprednisolone was administered three times per day instead. The recipients also received 250 mg ganciclovir intravenously and 1 g SMZ-TMP twice per day. Sporanox (itraconazole) and Diflucan (fluconazole) were administered to prevent mixed infection. CT scans were performed every four days to monitor the conditions of the patients. If the condition improved, the dosage of methylprednisolone was gradually reduced, while the dosage of immunosuppressants was gradually increased. However, if the condition of the patient deteriorated, oxygen inhalation or ventilatory support was provided. Furthermore, the methylprednisolone dose was increased to 80 mg and administered three times per day and all oral immunosuppressants were withdrawn.

For the recipients that were diagnosed with CMV pneumonia in Group I, all immunosuppressants, including tacrolimus and ciclosporin A, were withdrawn. Twice per day, the recipients were administered 80 mg methylprednisolone, 250 mg ganciclovir intravenously and 1 g SMZ-TMP. Sporanox (itraconazole) and Diflucan (fluconazole) were also administered to prevent mixed infection. During the hospital stay, patients received intensive supportive care, including early administration of albumin and γ-globulin. Similarly to Group S, CT scans were performed every four days. If the condition of the patient improved, the dosage of methylprednisolone was gradually reduced, while the dosage of immunosuppressants was gradually increased. However, if the condition of the patient deteriorated, the methylprednisolone dose was increased to 80 mg and administered three times per day. In addition, based on the results of blood gas analysis, the patients were provided with continuous oxygen inhalation via a mask or a noninvasive ventilatory support if required.

### Statistical analysis

Statistical analysis was performed using SPSS 13.0 (SPSS, Inc., Chicago, IL, USA). Quantitative data are presented as the mean ± SD. Numerical data were analyzed with the coefficient of variation (CV; calculated as SD/mean × 100%). The results between the groups were compared with the χ^2^ test. P<0.05 was considered to indicate a statistically significant difference.

## Results

### CMV pneumonia

Characteristics of patients in Group S and Group I were comparable as there were no statistically significant differences with regard to age, gender, weight, immunosuppressant regimen, average hospital stay and rejection rate during the treatments (Table I; P>0.05). A total of 38 kidney recipients developed CMV pneumonia in Group S, which was a significantly higher incidence rate when compared with Group I (45, 10.34%; P<0.05). In addition, patients in Group I exhibited a higher cure/recovery rate when compared with Group S (Group I, 100%; Group S, 92.11%; P<0.05). The mortality rate directly from CMV pneumonia was 2.41% (2/83) and the two cases were in Group S. An additional case finally ceased treatment (Group S, Table II).

The CV for treatment duration in Group I was 0.45, which was smaller as compared with Group S (0.50), indicating that the treatments in Group I were relatively more stable and effective. Notably, patients in Group I experienced an average duration of 156.78 days between kidney transplantation and the onset of primary CMV pneumonia, which was longer compared with Group S (93.00 days) and exhibited a delay of onset of CMV pneumonia (Table III).

### Association between CMV pneumonia and antibody induction therapy

A total of 573 patients underwent allograft renal transplantation between January 2008 and December 2011. Among these patients, 183 patients received antibody induction therapy with basiliximab or ATG. The results demonstrated that among the 83 patients that developed CMV pneumonia following kidney transplantation, 30 patients received antibody induction therapy. In Group S, 16 patients that received antibody induction therapy developed CMV pneumonia (16/47=34.04%), while 22 patients that did not receive antibody induction therapy developed CMV pneumonia (22/91=24.18%). The former was significantly higher than the later (Table IV; P<0.05). However, in Group I, there was no statistically significant difference in the incidence rate of CMV pneumonia between the patients that received and did not receive antibody induction therapy (Table IV; P>0.05). In addition, patients in Group I exhibited a lower overall incidence rate of CMV pneumonia when compared with Group S, regardless of antibody induction therapy administration (Table IV).

## Discussion

During active infection, CMV rapidly duplicates in various cells and tissues and spreads by cell-to-cell transmission, causing serious infection and intensifying complications in individuals with weakened immune systems. In the present study, the anti-CMV effects of ganciclovir were compared between patients administered intravenous ganciclovir for 2 weeks during the hospital stay in Group S and patients administered oral ganciclovir for 3 months during the post-discharge period in Group I. Oral ganciclovir treatment time in the present study was longer when compared with other studies (3,4). The results indicated that a longer duration of antiviral therapy with oral ganciclovir markedly reduced the incidence of CMV infection.

Reduction or even withdrawal of immunosuppressive drugs, such as CNIs, at an early stage of CMV pneumonia is often an important prophylaxis method for kidney recipients. In the present study, patients with CMV pneumonia in Group S were administered a reduced dose of tacrolimus and ciclosporin A by one third and a reduced dose of MMF by a half, whereas all immunosuppressants were withdrawn in Group I. The results indicated that CMV infection was unable to be controlled in five recipients in Group S until the immunosuppressant treatment was completely stopped. By contrast, all the patients in Group I exhibited an improvement to a certain extent at day 4 of treatment, as shown through CT scans. Therefore, withdrawal of immunosuppressants may facilitate the control of CMV infection.

Glucocorticoids are used to treat CMV pneumontitis and prevent acute kidney rejection (13). In the present retrospective study, the patients in the two groups were administered glucocorticoids. No patients exhibited adverse effects from the glucocorticoids. The results indicated that regular application of glucocorticoids is a safe and effective treatment for CMV pneumonitis. By contrast, the majority of kidney recipients had poor health due to long-term loss of renal function and toxicity from long-term use of immunosuppressants. Furthermore, patients with CMV pneumonia developed dyspnea and fever, which increased their energy consumption. Therefore, during the treatment period, nutritional support was required, including intravenous injection of γ-globulin and albumin, to correct hypoproteinemia. When the patients showed signs of short breath or dyspnea, oxygen saturation or the partial pressure of oxygen in the arterial blood (Pao_2_) were closely monitored. Oxygen inhalation or ventilatory support was provided at an early stage to correct hypoxemia when required, particularly when the Pao_2_ decreased.

In Group S, the incidence rate of CMV pneumonia in recipients that received induction therapy was higher compared with those that did not receive the induction therapy. However, in Group I, the incidence rate of CMV pneumonia did not increase with the administration of induction therapy (P<0.05). Therefore, a longer duration of prophylaxis was associated with a reduced risk of CMV infection in transplant recipients.

CMV infection typically occurs between 3 and 4 months following transplantation. In the current study, patients with CMV pneumonia in Group I experienced an average duration of 156.78 days between kidney transplantation and the onset of CMV pneumonia, thereby exhibiting a delay of onset of CMV pneumonia. This possibly results from proper prophylaxis (3-month course of oral ganciclovir and SMZ-TMP), improved hospital wards and good follow-up care. Longer duration between kidney transplantation and the onset of CMV pneumonia was reported in the present study for the first time.

In conclusion, the present study demonstrated that a longer duration of oral antiviral drugs, including ganciclovir, may effectively reduce the risk of CMV pneumonia in kidney transplant recipients. Several methods in the current study were shown to be effective, rapid and safe in controlling CMV pneumonia. These included early withdrawal of immunosuppressants, regular use of glucocorticosteroids and careful supportive treatment, including intravenous nutrition and oxygen inhalation. Furthermore, with proper prophylaxis, antibody induction therapy is not likely to increase the risk of CMV pneumonia in kidney recipients.

## Figures and Tables

**Table I:** Characteristics of the patients diagnosed with CMV pneumonia in Group S and Group I.

Patient characteristics	Group S	Group I
Kidney transplant recipients, n	138	435
CMV pneumonia cases, n	38	45
Average age of recipients with CMV pneumonia, years	41.25±17.36	39.94±18.02
Gender of recipients with CMV pneumonia, n		
Male	31	37
Female	7	8
Body weight of recipients with CMV pneumonia, kg	61.30±15.40	60.50±17.80
Warm ischemia time, min	7.4±4.8	7.0±5.5
Cold ischemia time, h	10.1±4.6	10.4±5.7
Patient immunosuppressant treatment, n		
Ciclosporin A + MMF + prednisone	16	17
Tacrolimus + MMF + prednisone	22	28
Rejection rate during treatment, %	7.89	6.67

CMV, cytomegalovirus; MMF, mycophenolate mofetil; Group S, standard treatment; Group I, improved treatment.

**Table II:** Comparison of anti-CMV effects between Group S and Group I.

Anti-CMV effects	Group S	Group I
Incidence rate of CMV pneumonia, %	27.54^a^	10.34
Average hospital stay, days	22.58±11.33	25.26±9.47
Cure/recovery rate, %	92.11^b^	100
Mortality rate, %	5.26^c^	0

a P<0.05, vs. Group I;

b P<0.05, vs. Group I;

c P<0.05, vs. Group I.

CMV, cytomegalovirus; Group S, standard treatment; Group I, improved treatment.

**Table III:** Comparison of treatment duration and onset time of CMV pneumonia between Group S and Group I.

	Treatment duration, days			Duration between transplantation and the first onset of CMV pneumonia, days		
		
Group	Mean	SD	CV	Mean	SD	CV
Group S	22.58	11.33	0.50	93.00	116.69	1.25
Group I	25.26	11.47	0.45	156.78	103.02	0.66

CMV, cytomegalovirus; CV, coefficient of variation; Group S, standard treatment; Group I, improved treatment.

**Table IV:** Association between CMV pneumonia and antibody induction therapy.

Parameter	Group S	Group I
Kidney transplant recipients, n	138	435
Recipients receiving antibody induction, n	47	136
Recipients not receiving antibody induction, n	91	299
Incidence of CMV pneumonia, n (%)	38 (27.54)^a^	45 (10.34)
Recipients that received antibody induction and developed CMV pneumonia, n (%)	16 (34.04)	14 (10.29)
Recipients that did not receive antibody induction and developed CMV pneumonia, n (%)	22 (24.18)	31 (10.37)

a P<0.05, vs. Group I.

CMV, cytomegalovirus; Group S, standard treatment; Group I, improved treatment.
